# Exploring gender disparities in the disease and economic tobacco-attributable burden in Latin America

**DOI:** 10.3389/fpubh.2023.1321319

**Published:** 2024-02-12

**Authors:** Andrea Alcaraz, Elena Lazo, Agustín Casarini, Federico Rodriguez-Cairoli, Federico Augustovski, Ariel Bardach, Lucas Perelli, Alfredo Palacios, Andrés Pichon-Riviere, Natalia Espinola

**Affiliations:** ^1^Instituto de Efectividad Clínica y Sanitaria (IECS), Institute for Clinical Efectiveness and Health Policy (IECS), Buenos Aires, Argentina; ^2^Consejo Nacional de Investigaciones Científicas y Técnicas (CONICET), Buenos Aires, Argentina

**Keywords:** gender, burden of disease, tobacco use, health disparities, Latin America

## Abstract

**Introduction:**

Tobacco use has significant health consequences in Latin America, and while studies have examined the overall impact, the gender-specific effects have not been thoroughly researched. Understanding these differences is crucial for effective tobacco control policies. The objective of this study was to explore the differences in tobacco-attributable disease and economic burden between men and women in Argentina, Brazil, Chile, Colombia, Costa Rica, Ecuador, Mexico, and Peru.

**Methods:**

We used a previously validated economic model to quantify the impact of tobacco-related illnesses, including morbidity, mortality, healthcare costs, productivity losses, informal care expenses, and DALYs, by gender and age. We utilized data from national surveys, records, studies, and expert opinions to populate the model.

**Results:**

In 2020, there were 351,000 smoking-attributable deaths. Men accounted for 69% and women 31%. Ecuador and Mexico had the highest male-to-female death ratio, while Peru and Chile had the smallest disparities. 2.3 million tobacco-related disease events occurred, with 65% in men and 35% in women. Ecuador and Mexico had higher disease rates among men, while Peru had a more balanced ratio. Regarding DALYs, men lost 6.3 million due to tobacco, while women lost 3.3 million, primarily from COPD, cardiovascular disease, and cancer. Brazil and Mexico had the highest DALY losses for both genders. Costa Rica had a lower male-to-female tobacco use prevalence ratio but ranked second in deaths, disease events, and DALYs attributed to tobacco. Colombia had a unique pattern with a male-to-female death ratio of 2.08 but a higher ratio for disease events. The health systems spent $22.8 billion to treat tobacco-attributable diseases, with a male-to-female cost ratio 2.15. Ecuador showed the greatest gender cost difference, while Peru had the lowest. Productivity loss due to tobacco was $16.2 billion, with Ecuador and Mexico exhibiting the highest gender disparities and Peru the lowest. Informal care costs amounted to $10.8 billion, with men incurring higher costs in Ecuador, Costa Rica, and Mexico.

**Discussion:**

Tobacco causes significant health and economic burdens in Latin America, with gender-based differences. There is a need for gender-disaggregated data to improve tobacco control policies.

## Introduction

Tobacco use is the leading preventable cause of disease and premature death worldwide (1). In the Americas, the prevalence of smoking is higher for men (21.3%) than for women (11.3%) in 2020 (2, 3). Data shows that the trend of tobacco use is declining; however, tobacco use among women is decreasing at a much slower rate than among men (3). At present, the difference in smoking prevalence between males and females is smallest in the Americas and Europe when compared to other regions of the world (3, 4). In addition, there is evidence of a high prevalence of female smoking among adolescents aged 13–15 years, even at the European level (2, 3).

A significant body of evidence shows the differences between men and women in tobacco use and how these differences could contribute to several diseases (5, 6). Recent research has revealed that female smokers face a significantly higher risk of acute coronary syndrome with obstructive coronary artery disease compared to their male counterparts (7, 8). In contrast, smoking has been associated with intracranial calcifications of the internal carotid artery in men with ischemic stroke, while hypertension and diabetes were identified as strong risk factors in women (9) When it comes to lung cancer, some studies indicate that women may be more susceptible to lung carcinogens than men and may develop cancer even with lower levels of cigarette use (10). On the other hand, although chronic obstructive pulmonary disease (COPD) has long been considered a male disease, several studies have shown that women report more symptoms of dyspnea, cough, and decreased forced expiratory volume, even when they have a similar pack-years history of smoking (11).

The differential negative impact to tobacco uses by gender goes beyond health outcomes. The chronic and globally progressive nature of tobacco-attributable diseases is associated with a continuous increase in the utilization of healthcare-related resources, impacting not only patients and their families but also society as a whole (12, 13). In Latin America in particular, smoking generates $34 billion in direct medical costs each year, representing a significant portion of that subregion’s healthcare budgets (14). In addition, there are studies that suggest that tobacco use has a significant impact on social costs, which could further deepen gender gaps if we look behind the numbers (15–17). This situation may have an unequal impact on financial protection based on gender, due to disparities in earnings and labor opportunities between genders (18). It is well known that there is significant economic inequality in the region and, the efforts to reduce poverty have not equally benefited men and women, nor have they progressed at the same pace. In 2021, according to the femininity index for every 100 men living in poor households in the region, there were 116 women facing a similar situation (19). Furthermore, socially prescribed gender roles assign women as the main ones responsible for family care. It is estimated that approximately 90% of women in Latin American countries engage in unpaid health care and household chores, dedicating twice as much time to these family responsibilities compared to men (20). These aspects significantly expand the scope of understanding the gender-based implications of social costs associated with tobacco, emphasizing their importance in informing policy decisions, as well as, the understanding of the differential effects of tobacco control policies by subpopulations (21–23).

For a long time, tobacco control overlooked the importance of analyzing tobacco use from a gender perspective. This can be attributed to the limited attention given to integrating gender considerations in research, policies, and programs, thus impeding progress in this domain (24). Recently, the World Health Organization (WHO) and the parties to the Framework Convention on Tobacco Control recognized the imperative need of recommendations to address gender-specific risks associated with tobacco. These recommendations encompass a range of actions such as augmenting funding for gender-specific research and advocacy, using sex-disaggregated data, implementing affordable tobacco control programs, addressing the connection between women’s liberation and tobacco use, and focusing on education for women and girls. These measures are crucial for effectively tackling the globalization of smoking-related challenges (24, 25).

The estimates and projections for the entire region of the Americas carried out by the WHO indicate that the association of smoking is greater in men (2, 26), which can also be reflected from the economic perspective. However, these data often mask large differences in men and women between and within countries. Therefore, disease burden and cost analyses are valuable in informing the diverse impact of tobacco-attributable diseases and thus helping decision makers to allocate resources and implement tobacco control measures and public policies at the optimal time.

The aim of this study was to explore the differences between men and women in the health and economic burden attributable to smoking in Argentina, Brazil, Chile, Colombia, Costa Rica, Ecuador, Mexico, and Peru. These countries lead the economic income of the region and represent 80% of the Latin American population.

## Methods

**Figure 1:**
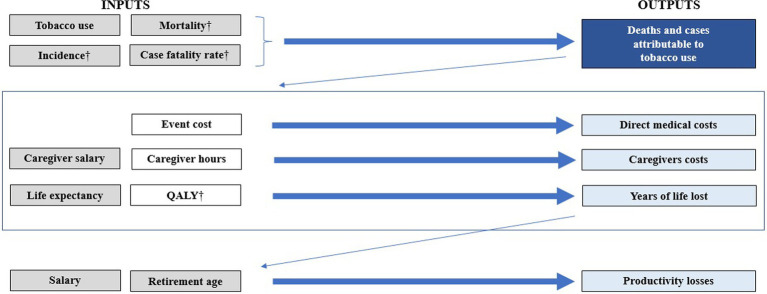
The burden of disease model structure and availability of data by sex. In gray the inputs that are differentiated between men and women. In dark blue the results that fully contemplate the differentiation between men and women. In clear light blue those who contemplate it at least partially. †Of related diseases with tobacco use. QALY, quality-adjusted life-year.

This study is based on an economic model already published and previously validated in 13 countries. The economic model is a state transition or probabilistic Markov microsimulation (first-order Monte Carlo technique) that considers the natural history, direct medical costs, indirect costs, and, quality losses associated with the main tobacco-attributable diseases (coronary and noncoronary heart disease, cerebrovascular disease, COPD, pneumonia, influenza, lung cancer, and nine other neoplasms) (27, 28). Its characteristics, components, validation, and applications are described in previous publications (16, 27, 29–31). In the model, adult people (35 years and over) are followed in hypothetical cohorts, and individual annual risks of disease incidence, disease progression, and death are estimated based on demographic characteristics of the population, smoking status, previous clinical conditions, and underlying risk equations to present aggregated results on mortality, disease events, quality of life, health care costs and, indirect costs (lost productivity). It is relevant to emphasize that the hypothetical cohort was chosen to commence at the age of 35 because it is from this age onwards that chronic diseases related to smoking begin to be observed. Furthermore, the hypothetical cohort was selected to represent the adult population of Latin America, which has an average age of around 35 years. This decision not only mirrors the epidemiological reality but also enables a more precise capture of the impacts of this habit on health as the population ages. Additionally, the selection of this hypothetical cohort was made to accurately depict the adult population of Latin America, where the average age is approximately 35 years. This approach ensures that the model’s results are more applicable and representative for the region by considering the specific demographic characteristics of the population under study (32). Most of the data is disaggregated by sex and the risk of the events is estimated from the baseline risk in non-smokers multiplied by the age, sex, and condition-specific relative risks (RR) for smokers and ex-smokers (33). The main characteristics of the model are shown in Figure 1.

### Information sources

Data to populate the model were obtained from a literature review that used MEDLINE, LILACS, Embase, EconLit, Google (for gray literature), and Google Scholar. Public statistics and country-representative surveys were the main sources of information on demographics, mortality rates, and smoking prevalence by sex, and age. Research teams from participating countries provided additional information from local sources on civil registrations, vital statistics, and hospital databases, and validated the epidemiological parameters used. The data included is available in Table 1.

**Table 1:** Annual burden of mortality, disease incidence and DALYs attributable to tobacco by sex and country for 2020.

Country	Chile	Peru	Brazil	Argentina	Costa Rica	Mexico	Colombia	Ecuador	Total
Total population									
Over 35 years old	9,771,671	13,390,445	98,134,446	20,404,023	2,299,805	51,796,845	21,977,761	6,335,146	236,441,488
Men	48%	49%	47%	47%	49%	47%	47%	49%	48%
Woman	52%	51%	53%	53%	51%	53%	53%	51%	52%
Ratio	0.92	0.96	0.89	0.89	0.96	0.89	0.89	0.96	0.92
Tobacco use									
Men	26%	29%	16%	16%	10%	21%	11%	15%	18%
Woman	19%	18%	9%	7%	4%	6%	3%	3%	9%
Ratio	1.37	1.61	1.78	2.29	2.50	3.50	3.67	5.00	2.09
Death attributable to tobacco use									
Men	11,594	12,853	110,961	31,023	1,719	48,141	20,500	5,447	242,238
Women	7,496	9,503	51,023	13,776	460	15,136	9,859	1,377	108,630
Ratio	1.55	1.35	2.17	2.25	3.74	3.18	2.08	3.96	2.23
Diseases events atributable to tobacco use									
Men	78,938	75,307	739,425	149,471	13,476	355,346	114,810	44,499	1,571,272
Women	53,290	61,343	421,470	83,042	3,643	105,985	63,509	10,086	802,368
Ratio	1.48	1.23	1.75	1.80	3.70	3.35	1.81	4.41	1.96
DALYs atributable to tobacco use									
Men	320,859	359,037	3,025,355	744,303	46,726	1,188,361	504,249	146,530	6,335,421
Women	223,393	292,910	1,632,548	399,061	14,230	411,942	255,428	41,808	3,271,320
Ratio	1.44	1.23	1.85	1.87	3.28	2.88	1.97	3.50	1.94

The direct medical costs associated with tobacco-related diseases were estimated using a mixed-method approach based on data availability. In cases where cost data was available, the micro cost method was applied, which involves calculating the cost of resources required for diagnosis, treatment, and follow-up and weighting them by usage rates for each disease related to tobacco use. However, for certain diseases, expert consultations and Delphi panels were employed to estimate the cost of treatment. Our report presents the average direct medical costs from a third-party payer perspective. Macroeconomic parameters, such as gross domestic product (GDP) and health expenditure, were extracted from data banks of multilateral organizations. The costs of labor productivity loss attributable to tobacco use were estimated considering the premature death of working-age individuals and the decrease in individuals’ labor productivity due to a health condition (absenteeism). To estimate the cost component associated with premature death, we applied the Value of a Statistical Life formula (34). For the absenteeism cost component, we adopted an indirect estimation criterion, assuming that individuals’ work productivity decreased proportionally to the reduction of quality of life attributed to that condition (35). To estimate both cost components, we calculated individuals’ labor income (by sex) through a Mincer equation (36) using representative household surveys, and the legal retirement age by sex in each country (37). For further details see Pinto et al. (16). The costs of time use of informal caregivers (those who provide care to family members without receiving remuneration or economic compensation for it) was estimated through the proxy good approach using information from Espinola et al. (20, 38). We estimated the costs in local currency units. Then, we converted to 2020 US dollars (USD) using the average exchange rates for each local currency, which were obtained from the web page of each Central Bank. (39–45)

The epidemiological data utilized to populate the model were gathered for the year 2020, and the key inputs are outlined in Table 1 and the Supplementary material. Notably, we observed an imbalance in the gender distribution among individuals over 35 years of age in 2020 across the eight countries under study. Despite there being more women than men in this age group, tobacco use is more prevalent among men, with variations observed among countries. For instance, in Ecuador, the ratio is five men to every woman using tobacco, whereas in Chile, the ratio is 1.34 men to every woman (Table 1). On another note, when examining the data on the relative risks of smokers versus non-smokers, it becomes evident that female smokers generally exhibit a higher relative risk than men, except for lung cancer, where men have almost twice the relative risk of women. Conversely, the data on the relative risks of smokers versus former smokers indicates that male smokers tend to have a higher relative risk compared to female smokers, except for stroke (1/0.89, a 1.25-fold lower risk) and COPD, where the risk is similar. As for the ‘basal mortality rate due to diseases associated with tobacco use,’ in most countries, mortality is higher in men than in women. This rate represents overall mortality before factoring in additional risk factors and specifically pertains to mortality caused by diseases associated with tobacco use. (32) However, for certain conditions such as other heart and cardiovascular diseases (Chile, Costa Rica, Colombia, Ecuador, and Mexico), stroke (Chile, Costa Rica, Colombia, Mexico, and Peru), and pneumonia/influenza (Argentina, Brazil, Chile, and Colombia), there is higher mortality in women than in men. Additionally, there is greater mortality from lung cancer and AMI in women than in men in Colombia (Supplementary Tables S1, S2).

### Estimation of the smoking-attributable disease burden

The main outcomes of the model were deaths, disease events, healthy years of life lost due to premature death and disability, and disease costs by sex in each country. The disease burden was estimated as the difference in outcomes between the results predicted by the model for each country under current smoking prevalence and a hypothetical cohort of individuals who never smoked. Passive smoking and perinatal effects were estimated to impose an additional burden of 13·6% (men) and 12% (women) (46).

### Model calibration and validation process

Disease-specific mortality rates for sex were compared to local statistics in each country. Predicted rates within 10% of references were considered acceptable. With larger deviations, risk equations were calibrated. The model was externally validated against other epidemiological and clinical studies not used for equation estimation and development.

## Results

In Table 1, we present the results of the tobacco-attributable disease burden by sex in 2020. Overall, the study estimated a total of 351,000 smoking-attributable deaths in the eight countries. Among these deaths, approximately 69% were of men, while 31% were of women. This resulted in a male-to-female death ratio of 2.23, indicating that men experienced more than twice as many deaths compared to women due to tobacco use. Among the countries with the highest male–female mortality rates are Ecuador and Mexico, with ratios of 3.96 and 3.18, respectively. On the other hand, Peru and Chile had the smallest disparities in deaths between men and women, with ratios of 1.35 and 1.55, respectively. The variations observed between countries can be attributed to differences in the prevalence of smoking between men and women, although the relationship is not strictly linear.

The model projected an annual estimate of 2.3 million disease events directly related to tobacco use. This distribution between genders reflects the patterns of death observed. Among these, about 1.5 million (65%) were projected to occur in men, while approximately 800,000 (35%) were estimated to occur in women. Ecuador and Mexico exhibited more tobacco-attributable disease events per year among men compared to women. Ecuador, for example, showed a ratio of 4.41 disease events in men for each one in women (Table 2).

**Table 2:** Annual burden of mortality and disease incidence attributable to tobacco by sex, country, and specific diseases for 2020.

Country	Chile	Peru	Brazil	Argentina	Costa Rica	Mexico	Colombia	Ecuador	Total
Deaths									
*Cardiovascular disease*									
Men	2,042	1,493	24,271	7,853	433	15,580	5,357	1,222	58,251
Women	944	759	8,908	2,193	86	3,950	2,595	229	19,664
Ratio	2.16	1.97	2.72	3.58	5.03	3.94	2.06	5.34	2.96
*Stroke*									
Men	772	912	6,159	1,371	69	2,996	1,057	360	13,696
Women	472	626	3,882	804	25	1,097	598	111	7,615
Ratio	1.64	1.46	1.59	1.71	2.76	2.73	1.77	3.24	1.80
*Lung cancer*									
Men	1,986	1,324	15,903	6,018	198	3,647	2,891	484	32,451
Women	1,217	1,097	8,540	2,573	56	1,475	1,446	196	16,600
Ratio	1.63	1.21	1.86	2.34	3.54	2.47	2.00	2.47	1.95
*Pneumonia/influenza*									
Men	425	1,684	8,882	2,683	83	3,232	594	438	18,021
Women	268	1,190	3,319	1,245	14	848	189	76	7,149
Ratio	1.59	1.42	2.68	2.16	5.93	3.81	3.14	5.76	2.52
Other cancers*									
Men	2,149	1,991	20,757	4,325	278	4,680	2,790	739	37,709
Women	891	1,098	4,926	1,427	54	1,169	794	159	10,518
Ratio	2.41	1.81	4.21	3.03	5.15	4.00	3.51	4.65	3.59
*Passive smoking*									
Men	1,388	1,539	13,284	3,714	206	5,763	2,454	652	29,000
Women	803	1,018	5,467	1,476	49	1,622	1,056	148	11,639
Ratio	1.73	1.51	2.43	2.52	4.20	3.55	2.32	4.41	2.49
*COPD*									
Men	2,832	3,910	21,705	5,059	452	12,243	5,357	1,552	53,110
Women	2,901	3,715	15,981	4,058	176	4,975	3,181	458	35,445
Ratio	0.98	1.05	1.36	1.25	2.57	2.46	1.68	3.39	1.50
Diseases events									
*Cardiovascular disease*									
Men	29,325	11,986	335,780	51,211	6,879	145,769	38,189	17,817	636,956
Women	13,569	7,306	157,284	16,324	1,047	25,337	20,812	2,280	243,959
Ratio	2.16	1.64	2.13	3.14	6.57	5.75	1.83	7.81	2.61
*Stroke*									
Men	8,001	6,006	31,618	7,355	269	25,669	10,156	2,988	92,062
Women	4,579	4,649	21,119	4,049	103	9,058	6,480	857	50,894
Ratio	1.75	1.29	1.50	1.82	2.61	2.83	1.57	3.49	1.81
*Lung cancer*									
Men	2,219	1,455	16,940	6,557	254	4,224	3,088	527	35,264
Women	1,458	1,275	9,186	2,981	68	1,836	1,565	228	18,597
Ratio	1.52	1.14	1.84	2.20	3.74	2.30	1.97	2.31	1.90
*Pneumonia/influenza*									
Men	3,444	13,385	77,596	20,235	581	32,518	4,764	3,830	156,353
Women	2,067	10,784	37,382	12,452	192	11,427	2,072	918	77,294
Ratio	1.67	1.24	2.08	1.63	3.03	2.85	2.30	4.17	2.02
Other cancers*									
Men	3,448	3,056	32,411	7,433	478	7,118	4,357	1,069	59,370
Women	1,398	1,789	7,850	2,221	90	1,884	1,231	253	16,716
Ratio	2.47	1.71	4.13	3.35	5.31	3.78	3.54	4.23	3.55
*COPD*									
Men	32,501	39,419	245,080	56,680	5,015	140,048	54,256	18,268	591,267
Women	30,219	35,540	188,649	45,015	2,143	56,443	31,349	5,550	394,908
Ratio	1.08	1.11	1.30	1.26	2.34	2.48	1.73	3.29	1.50

In terms of disability-adjusted life years (DALYs), the results highlight those men experienced a loss of 6.3 million DALYs due to tobacco-related premature mortality and disability, while women registered half of that figure with 3.3 million. The main drivers of these numbers were COPD, cardiovascular disease, and cancer. Brazil and Mexico emerged as the countries with the highest DALY loss for both sexes, underscoring the profound impact of tobacco-related health outcomes (Supplementary Table S3).

**Figure 2:**
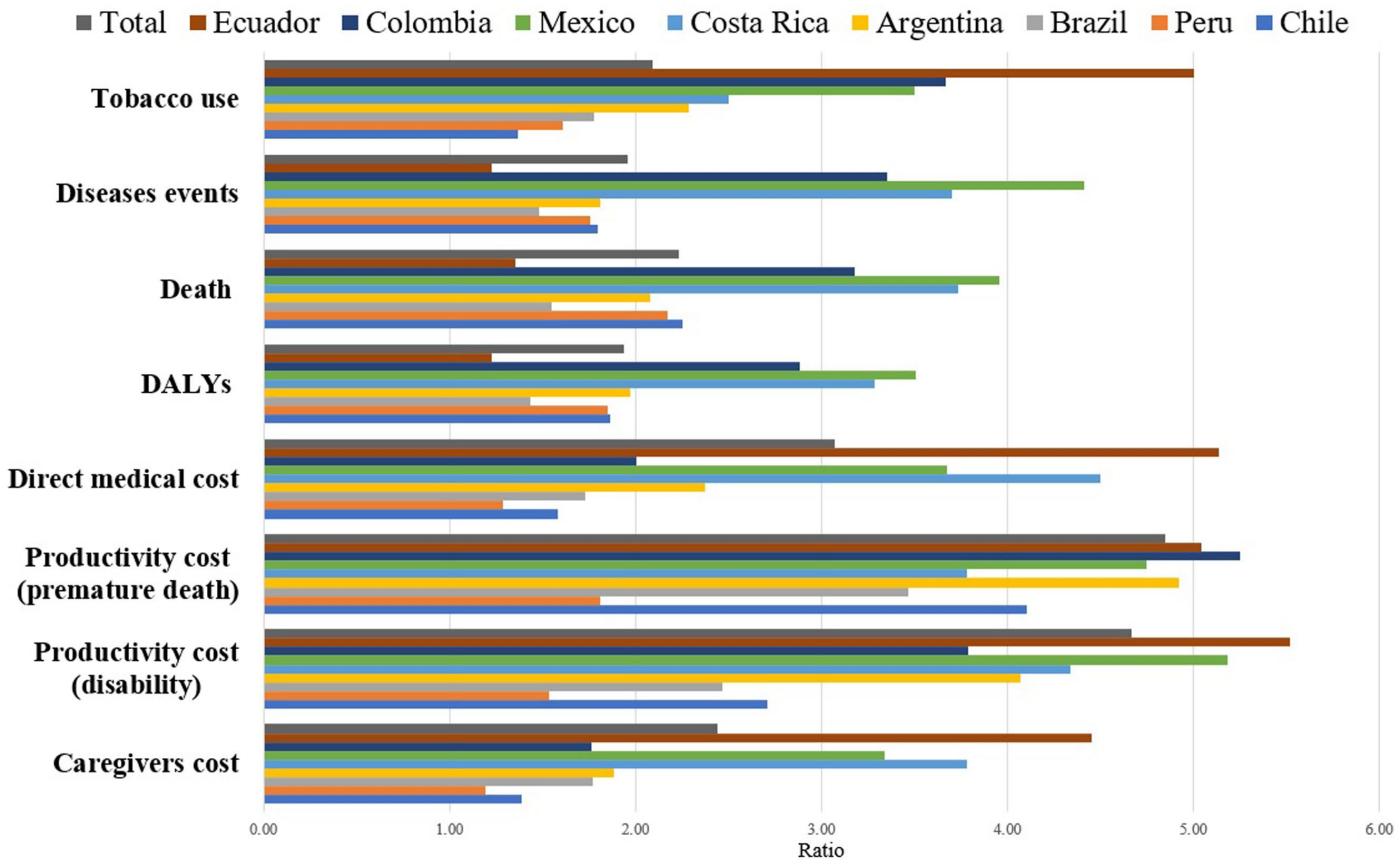
Ratios of men/women in tobacco use and the associated disease and economic burdens by country for the year 2020.

Particularly interesting is the case of Costa Rica, where the prevalence ratio of tobacco use between men and women (2.5) appears lower compared to Ecuador (5) and Mexico (3.5). However, it stands out with the second-highest ratios in terms of deaths, disease events, and DALYs attributable to tobacco, positioning itself between Ecuador and Mexico. Conversely, Colombia presents an interesting scenario with a tobacco use prevalence of 11% among men and 3% among women, resulting in a ratio of 3.67. Its male-to-female death ratio attributed to tobacco is 2.08, marking it as the third-lowest ratio among the eight countries. Nonetheless, when it comes to tobacco-attributed disease occurrences, Colombia demonstrates a higher ratio compared to the other nations (Figure 2).

Additionally, Table 3 shows the results of the economic burden attributable to tobacco by sex in 2020. We estimated that the health systems in these eight countries spent $22.8 billion in direct medical costs due to tobacco use. Of this total, $15.5 billion was spent by men and $7.2 billion by women. This represents a male-to-female ratio of 2.15. Ecuador had the greatest disparity in costs between men and women, with $5.14 spent on men for every dollar spent on women, while Peru was the country with the lowest difference in cost between men and women (1.29 dollars for every dollar in women).

**Table 3:** Annual economic burden attributable to tobacco by sex and country for 2020 (USD millions).

Country	Chile	Perú	Brazil	Argentina	Costa Rica	México	Colombia	Ecuador	TOTAL
Direct medical cost									
Men	1,198,735,774	683,401,917	5,921,380,002	1,957,452,356	233,312,455	4,222,520,742	780,666,475	548,870,177	15,546,339,898
Women	756,254,016	531,802,466	3,426,030,681	823,617,858	51,824,272	1,148,300,627	388,961,213	106,865,913	7,233,657,046
Ratio	1.59	1.29	1.73	2.38	4.50	3.68	2.01	5.14	2.15
Productivity cost (early death)									
Men	327,300,700	198,124,120	2,694,105,227	556,032,944	37,098,478	878,008,000	205,570,417	100,504,334	4,996,744,219
Women	79,763,464	109,304,663	777,722,681	112,939,471	9,810,365	184,896,939	39,173,112	19,917,657	1,333,528,352
Ratio	4.10	1.81	3.46	4.92	3.78	4.75	5.25	5.05	3.75
Productivity cost (disability)									
Men	527,746,658	259,544,149	3,885,387,260	643,137,177	79,950,884	1,389,582,989	300,452,179	198,990,057	7,284,791,354
Women	194,640,187	168,784,352	1,572,989,227	157,970,662	18,419,472	268,211,161	79,372,283	36,078,400	2,496,465,743
Ratio	2.71	1.54	2.47	4.07	4.34	5.18	3.79	5.52	2.92
Caregivers cost									
Men	655,832,930	380,948,275	3,847,047,539	697,604,326	79,106,691	710,139,143	331,215,745	254,939,143	6,956,833,791
Women	471,732,409	319,194,077	2,176,609,904	370,288,995	20,931,537	212,562,854	187,788,713	57,282,072	3,816,390,560
Ratio	1.39	1.19	1.77	1.88	3.78	3.34	1.76	4.45	1.82

The economic burden of tobacco-attributable productivity loss was estimated at $16.2 billion, with $12.3 billion in men and $3.9 billion in women. Among the analyzed cost categories, this cost exhibited the most significant difference by sex, consistently observed across countries. Ecuador and Mexico presented the greatest lost productivity costs, with 5.3 billion and 5.0 billion dollars lost in productivity in men for every dollar lost in productivity in women, respectively. Conversely, Peru has the lowest ratio of productivity loss costs for men to women among the countries examined. Informal care costs represented an additional burden of $10.8 billion, with $6.9 billion for caring for a man and $3.9 billion for caring for a woman. This means that men generate higher informal care costs than women. These differences are particularly marked in Ecuador, Costa Rica, and Mexico, where $4.45, $3.78, and $3.34 are spent to care for a man for every dollar spent to care for a woman, respectively.

## Discussion

Despite WHO estimates indicating a decrease in the prevalence of smoking in the Americas region, smoking remains one of the main causes of disease and economic burden in men and women, with large differences between and within countries. Our study estimates that almost a thousand people die every day because of tobacco use in these eight countries, and annually it causes more than 2 million disease events, including cardiovascular events, cancer, stroke, COPD, and other diseases. Men—being more likely than women to smoke—are also two times more likely to die from smoking, and to have disease events attributable to tobacco use. However, the study shows significant heterogeneity among the countries analyzed in the region. In some countries (Ecuador, Costa Rica, and Mexico) the deaths and cases in men are more than 3 times higher than women. Although this aspect is associated with a higher prevalence in the countries, the relationship is not completely linear.

The differences in tobacco prevalence rates and associated health burden by sex clearly illustrate that differences in tobacco use are not necessarily determined by sex differences in relation to the psychopharmacological properties of nicotine or other tobacco components, and that social opinions play an important role in determining the smoking rates, possibly related to differences in gender equality between countries. (47, 48) To such an extent that the WHO points out that in countries where women are more empowered, smoking rates for women are higher than those for men, regardless of income inequality. (48) Similarly, other social determinants, such as ethnic origin and socioeconomic position, play an important role during the first years of life, when health behaviors and risk factors are formed, up to adolescence and adulthood. (24, 49) Specifically, related to gender, an example would be the products designed by the tobacco industry, such as “light” cigarettes marketed specifically for women, they are smoked with greater intensity and have higher yields of nitrosamines, which is responsible for the increase in lung cancer in women. Another example would be the gender bias in the diagnosis of COPD, men are more likely to be diagnosed with COPD than women with the same symptoms, delaying their diagnosis. (47, 48) So we can mention that the differences between men and women in tobacco consumption and its impact are crossed by various socioeconomic and cultural factors that can explain the differences between countries, and within a country.

In addition, our study shows the gender difference in the economic burden attributable to tobacco use. In 2020, tobacco use causes $49.8 billion in economic losses in the eight countries. Of this total, direct costs accounted for 46%, productivity loss costs represented 33% and informal care represented 22%. The analysis by gender showed that the highest proportion of direct and indirect costs were generated by men. The greatest difference between men and women is observed in the costs of lost productivity, more than 3 times, even though the model does not consider differences according to participation in the labor market. This disparity not only correlates with increased mortality and morbidity rates among males but also underscores the disparity in labor income by gender. It is known that the prevalence of tobacco use is higher in lower-income households, (50) and society is experiencing a situation known as the feminization of poverty. (19) Therefore, the loss of employment or falling ill for a female head of household can be financially more catastrophic than for a male head of household. (51) On the other hand, the study reveals that 22% of the overall cost corresponds to expenditures on informal care. It is widely recognized that women predominantly assume the role of informal caregivers, further exacerbating their disadvantaged position. (20) Furthermore, when analyzing between countries, a significant heterogeneity in both direct and indirect costs can be observed, which further highlights the existing inequalities within the region.

The scope of the analysis was limited by data limitations. First, the information presented in this study is based on the available sex-disaggregated data, acknowledging its limitations. It is crucial to recognize that relying solely on sex-specific data may restrict a comprehensive understanding of the intricate relationship between gender dynamics and their impact on the research findings. Gender encompasses various social, cultural, and individual factors that extend beyond biological sex and can influence health outcomes and behaviors. Consequently, the interpretations and conclusions drawn from this analysis may not fully capture the nuanced interactions between gender and the subject matter under investigation. To enhance the depth and accuracy of future research, efforts should be made to collect and report both sex and gender-disaggregated data. Second, the data on health spending were not available by disease, sex, and age, so we were unable to perform a more detailed analysis of direct cost; although we use the best available information and apply a uniform and replicable method, the availability and quality of epidemiological and cost information in Latin America is heterogeneous, and this could have led to an underestimation or overestimation of the direct cost. Although all the main costs have been considered, the caregiver cost data is not disaggregated by sex, since we do not have information on the sex and age of the caregiver. However, there are several studies that show that informal care is mainly carried out by women. Third, although our study did not include all Latin American countries, the countries analyzed comprise 80% of the population and represent a diverse sample. Despite these limitations, our study provides a comprehensive and robust estimate of the health and financial burden of smoking in Latin America and shows a huge tobacco-attributable burden, which is likely a conservative estimate as data on the burden were not available from secondhand smoke.

This study makes visible the need to generate more evidence based on gender and diversity to develop more sensitive research focused on the differential needs of women who are affected by tobacco use. Understanding and incorporating both sex and gender perspectives in research design, data collection, analysis, and interpretation can lead to more comprehensive and accurate findings, ultimately contributing to better-informed policies and interventions. At the same time, we hope that this analysis expands future research, considering the contextual differences in the different countries of the Latin American region from a sensitive perspective that shows the differences in the burden of tobacco use between men and women and including advances in issues of gender in the region.

## Data availability statement

The original contributions presented in the study are included in the article/Supplementary material, further inquiries can be directed to the corresponding author.

## Author contributions

AA: Conceptualization, Investigation, Methodology, Project administration, Supervision, Validation, Visualization, Formal analysis, Writing – original draft. EL: Visualization, Writing – original draft, Formal analysis, Investigation, Methodology. AC: Data curation, Formal analysis, Visualization, Writing – review & editing. FR-C: Data curation, Writing – review & editing. FA: Conceptualization, Methodology, Supervision, Validation, Writing – review & editing. AB: Conceptualization, Methodology, Supervision, Validation, Writing – review & editing. LP: Data curation, Writing – review & editing, Visualization. AP: Conceptualization, Methodology, Validation, Writing – review & editing. AP-R: Conceptualization, Methodology, Supervision, Validation, Writing – review & editing. NE: Formal analysis, Methodology, Validation, Writing – review & editing, Conceptualization, Supervision.
